# Editorial Abstracts and Selections

**Published:** 1861

**Authors:** 


### Editorial Abstracts and Selections.

From a recent number of the *American Medical Times*, we learn that the Louisiana Legislature, through the influence of Dr. I. H. Stevens, has passed "an act relative to practitioners of medicine," by which it is made illegal for any one to practice in that State, without first making affidavit before a Justice of the Peace, said affidavit to be duly recorded by the Parish Recorder, that he has received the degree of Doctor of Medicine, from a regularly incorporated medical institution in America or Europe. Without such affidavit, no one may collect fees, but is liable to a penalty of $20 for every violation of the act.

Nitric Acid in Intermittent Fever.—As early as 1854, the anti-periodic properties of nitric acid had been presented to the profession, by Dr. George Mendenhall in the *Western Lancet;* and even prior to that time had been availed of by Dr. E. S. Bailey, of Indiana. A recent article in the *Maryland and Virginia Medical Journal*, by Professor Wm. A. Hammond, late U. S. Surgeon at Ft. Riley, Kansas, is again attracting the attention of the profession to its use for this purpose; and from it we condense the following: Forty-one cases, ten of the quotidian and thirty-one of the tertian type, form a table, the basis of a report made to the Surgeon-General. Of these, thirty-two were treated with nitric acid, and nine with quinine. Three cases, cured by nitric acid, had previously used quinine unsuccessfully; and two, cured by quinine, had been treated with nitric acid ineffectually, and in one other the acid was discontinued, on account of creating nausea, heart burn, etc. The average period of treatment was the same with each remedy—three days. The acid was given in doses of ten drops, three times per day, properly diluted with water; the quinine in eight grain doses, as often. Dr. Hammond says: Besides the fact that the nitric acid was equally successful with quinine in arresting the disease, the difference in the cost of the two articles is so greatly in favor of the former substance as to render it an object of importance to make its curative properties more widely known. ——Since the foregoing cases were treated, I have frequently employed nitric acid in the treatment of intermittent fever, and have rarely been disappointed in my expectations of its curative action. In fact, in simple, uncomplicated intermittent, I seldom have occasion to use anything else. ——In cases of enlargement of the spleen, consequent upon frequent attacks of the ague, the remedy in question, has, in my hands, proved very advantageous.

STEADINE.—The formula of a new and solid species of glycerine, soluble alike, to a certain extent, in oils and water, is thus given in the London *Jour. of Prac. Med. and Sur.* and *Pharm. Jour.* for Dec., 1860:

Lard,
Water, āā. ℥iiss
Soda, deprived of its carbonic acid by lime, grs. xv.

The soda is weighed and used dry. It should be melted in about four drachms of water; the lard is then gradually added alternately with the remaining water. The operation of this mixture is both swift and simple; in ten minutes four pounds of steadine may be prepared. This new adipose substance presents the appearance of a whitish, fatty compound, inodorous, insipid and intermediate between cerate and lard. It acquires firmness soon, and does not change as readily as lard under varying temperatures. It is claimed to be as serviceable as glycerine itself, for oils (medicated) and liniments; its ointments of metallic bases, oxides, sulphurets, iodides, salts, etc., remain unaltered; iodide of potassium pomade retains its whiteness and its iodine; insoluble powders mix with it promptly and accurately; it combines readily with, and is a better solvent for vegetable powders; soluble salts and aqueous extracts are easily and intimately associated with it—and in short, it would seem to be a very valuable adjunct to pharmacy.

Won't some of our druggists prepare some and report?

TURPENTINE AS AN ANÆSTHETIC.—Dr. John Wilmhurst, of the Cunard ship *Marathon*, writes to the London *Lancet*, that he believes he has discovered a new anæsthetic and valuable anodyne, in the rectified oil of turpentine. Dr. W. was accidentally led to use the oil, instead of chloroform, in a severe case of facial neuralgia; it was exhibited by sprinkling a few drops on a handkerchief and applying to the nostrils; a few inhalations, produced a gentle sleep, from which the patient awoke, free from pain, headache, or other unpleasant symptoms. He has used it subsequently in one or two operations, in cramps, renal calculus, and convulsions. He describes its effects as allaying nervous irritation, spasm, and pain, without deranging the heart's action, and producing a calm anæsthetic sleep.

GERMAN PHYSICIANS are using powdered malt as a nutrient for children, in Germany, with considerable confidence and success. Dr. Zieruck, of Berlin, after a comparative analysis, claims that it approximates more closely to the qualities of good human milk, containing a larger amount of phosphates and nitrogenized elements, with more assimilable hyro-carbonic elements in it, than either East India arrow-root, or the Racahout des Arabes.

CHLORATE OF POTASH IN PHTHISIS.—In connection with our obituary notice of the late Dr. Fountain, we give the following succinct, comprehensive *rēsumé* of his last paper, in the *American Medical Monthly*, on the subject of the researches which engaged his attention at the time of his death. We give it, also, as presenting a fair sample of the valuable exhaustive brevity with which Dr. Wells condenses the very pith and marrow of lengthy Magazine articles into the pages of his *Summary of Medical Science:*

"*Phthisis—Chlorate of Potash.*—Adopting as his motto a statement of Liebig, that 'oxygen is the leaden weight, or bent spring, which keeps the clock in motion; the inspirations being motions of the pendulum which regulate it,' Dr. Fountain, of Iowa, looks upon the therapeutical indications in tuberculosis and kindred diseases in a simply chemical light, and the treatment is practically reduced to the question, *by what*

*and in what manner* can we best supply to the system the oxygen which is demanded for the proper performance of its functions, and thereby counteract the deleterious influences resulting from the *imperfect aeration of the blood?*

Dr. Fountain details three cases, which, from all rational and physical signs present, must evidently be considered as tubercular, in which the treatment with chlorate of potash was followed by very satisfactory results. In one case the patient took half an ounce of the chlorate of potash daily for six weeks, and two drachms each day for the succeeding four weeks, and occasionally thereafter in similar quantities. At the time of writing, the man was actively engaged in business, in good strength and flesh, and apparently perfectly well.

The author remarks that, 'though the treatment was purely experimental, it was not empirical; for the chlorate of potash was given on the assumed principle of *conveying oxygen to the blood*, by which a portion of the lungs was expected to be relieved of their task; the vital power of the blood increased, rendered more capable to perform its functions, and by which tubercular deposits might be arrested, and absorption of those already formed promoted.' The author deduces the following conclusions from the case detailed:

1. The chlorate of potash can be given in large doses every day for a long period, without injury.
2. It aids the function of respiration by supplying the blood with oxygen.
3. It operates as a natural *tonic alterative* and *blood depurant*, by increasing the supply of that element which is the most active agent of nature in the chemical changes which take place in the laboratory of the human system."

---

Maxims of Military Surgery.—At a time when the entire press, professional and secular, is occupied with the one prevailing topic, and our periodical literature bristles with war-terms and phrases, as the country does with bayonets, we may be pardoned devoting considerable space to the subject of Military Surgery, and to any details of information incident to its study. The following extracts from Surgeon-General Stromeyer's *Maxims of Military Surgery*, originally published in two duodecimo volumes in 1858, and further amplified by an octavo supplement of pp. 150, at the commencement of the present year, will be found valuable as from the most recent contribution to the literature of this specialty. For them, as also for the comments included, we are indebted to the *N. A. Medico-Chirurgical Review:*

"The position of the military surgeon renders it scarcely possible to extend the science of war-surgery, unless he is placed in a hospital station. During a long peace, too, the impressions on the memory are weakened, and partially superseded by more recent occurrences. Dr. Stromeyer does ample justice to the ardent aspirations, so often cruelly disappointed, with which many young men enter the medical service of armies; but it is the duty of a philosopher and a man of business to contemplate things as they are, and to labor after, not what is best in the abstract, but what is obligatory and most useful in the existing state of things. The character of the experience gained, too, varies excessively; and examples are seen of army surgeons who have neglected to practice attendance on the wounded, and have first become familiar with bandaging on an actual field of battle.

In regard to their qualifications for this service, physicians fall into the following classes:—

1. Such as, by the variety of their talents, by their character, their experience in service, and their vigorous health, are competent to every duty.

2. Such as by their vigorous health, by peculiar adroitness in their intercourse with officers and men, are particularly qualified for service among marching troops.

3. Such as are peculiarly qualified for the treatment of in and out patients of hospitals.

4. Such as, from possessing the character of experienced surgeons and operators, are peculiarly qualified for the service of ambulances.

5. Those, whom a peculiar fitness for and inclination to administration, point out for the management of muster-rolls and accounts. A well organized army develops these men; but in a newly-raised body they belong to the exceptions.

6. Those, in fine, who should be got rid of as speedily as possible, as they do no particular honor to their station. On account of such individuals as these, where sudden and large augmentations are made in the numbers of the military medical body, the appointments may be made provisionally.

These qualifications, says Dr. Stroymeyer, cannot remain long concealed from an observing chief; and form, in his opinion, a much better basis for appointments in war than the over-estimated rolls of seniority. The latter consideration, when compared with fitness, should, in our author's opinion, be disregarded.

The author believes that in the north of Germany the service has been improved by the decay of the special schools for military medicine, and the assumption of competent men from the universities."

"Dr. Stromeyer is of opinion that the construction and management of hospitals should be regularly taught in universities by public courses, for the information of the young men in pursuit of medical honors; and that governments would then never experience any difficulty in selecting persons properly qualified to discharge these important duties.

In constructing military hospitals, part of the trouble is saved by the avoidance of the necessity of providing for both sexes. The idea has been relinquished, that it was desirable to assemble great numbers of patients with the same complaints in large wards; those more recently prepared not containing, in general, more than a dozen beds. This Dr. Stromeyer prefers, conceiving that the object of classification requires smaller rooms. In the General Hospital, at Hanover, there are rooms of three sizes; thirteen that contain sixteen beds each, sixteen for four beds, and eleven provided with only one or two apiece. It is difficult to estimate with confidence the relative proportions of mild cases and of the severe ones which any hospital is likely to receive; but by ascertaining the facts from similar institutions, it is not impossible to approximate to this result. The milder cases, for convenience, should be grouped in the larger rooms; but smaller ones are indispensable, from the necessity of separating the severe cases, and frequently of isolating them. The motives for this will sufficiently suggest themselves: silence; freedom from interruption; the necessities of peculiar cares, sometimes unpleasant to others; a peculiar state of the atmosphere, and of light; and lastly, the presence of those very ill or dying, with all the injurious effects upon the minds of those who are ill themselves, and often in a situation really, or in imagination similar. Dr. Stromeyer hence finds it necessary to intersperse the large wards with a sufficient number of smaller rooms to answer these purposes."

"The professor sums up the general impressions which his observations have left on his mind in the following dogmas or propositions, which he submits with great modesty to criticism:

1. The healing art is founded upon the natural history of disease. A good therapeutic method must follow the physiological processes which are disordered in the patient. This includes injuries which are the subject of surgery, and also poisoning.

2. There are no specific remedies. All that appear such should be explained physiologically.

3. Regulations of the mode of life of the patient is the most important problem which is laid before the physician, as it relates to the action of all the stimuli which support and modify life. This is equivalent to diet, in the original Hippocratic sense. (*Foes, sub verbo.*)

4. Medicine should never be given till the mode of life is previously regulated. To practice medicine in foul air, or during the use of improper food, is like making chemical investigations with impure reagents.

5. Drugs should be given when they are necessary or *euphemistic*, and when they are indicated. By the word which we have expressed in italics, we presume that the author means in good repute.

6. A good medicine must be one which will never add to any of the detriments which the patient is liable to suffer in the course of the disease. Such a remedy should be considered faulty or imperfect.

7. The natural history of the disease includes the groundwork of its existence; and this leads to the most noble problem submitted to physicians—the prevention of disease.

Military physicians are more frequently than others in a position to render services of this kind.

'These maxims,' says our author, 'characterize the true physician; and the reverse of them, the quack. Let them be to us words of counsel, beloved as the beautiful constellations that guide the ship through the ocean.'"

